# A potential link between endothelial function, cardiovascular risk, and metabolic syndrome in patients with Non-alcoholic fatty liver disease

**DOI:** 10.1186/1758-5996-6-109

**Published:** 2014-10-14

**Authors:** Muyesser Sayki Arslan, Sibel Turhan, Irem Dincer, Dilsa Mizrak, Demet Corapcioglu, Ramazan Idilman

**Affiliations:** Department of Internal Medicine, Ankara University, School of Medicine, Yeni Ziraat Mah. 656. sok. 22/4. Altındağ, Ankara, Turkey; Department of Cardiology, Ankara University, School of Medicine, Ankara, Turkey; Department of Endocrinology and Metabolism, Ankara University, School of Medicine, Ankara, Turkey; Department of Gastroenterology, Ankara University, School of Medicine, Ankara, Turkey

**Keywords:** Non-alcoholic fatty liver disease, Metabolic syndrome, Asymmetric dimethylarginine, Endothelial function, Cardiovascular risk

## Abstract

**Background:**

Asymmetric dimethylarginine (ADMA) is an endogenous competitive inhibitor of nitric oxide (NO) synthetase. Elevated ADMA reduces NO formation and is associated with endothelial dysfunction. The aims of this study were to evaluate endothelial function and the cardiovascular risk (CVR) profile in patients with non-alcoholic fatty liver disease (NAFLD), and to determine whether or not an association with metabolic syndrome (MS) increases these parameters.

**Methods:**

A total of 100 consecutive patients with NAFLD, who were seen in Liver Disease Outpatient clinic and 45 age- and sex-matched controls were included. Endothelial function was evaluated based on the serum ADMA level measured using a validated ELISA kit (DLD Diagnostika GMBH, Hamburg, Germany) and flow-mediated vasodilatation (FMV) measured via high-resolution external ultrasonography. The CVR profile was calculated according to the Framingham equation.

**Results:**

At baseline there weren’t any significant differences in brachial artery diameter between the NAFLD and control groups (3.7 ± 0.6 mm vs. 3.6 ± 0.6 mm, respectively). FMV and flow-independent vasodilatation in response to sublingual nitroglycerin did not differ between the NAFLD and control groups (mean: 16% ± 9.4% vs. 17.9% ± 12.4%, and 21.4% ± 14% vs. 17.8% ± 11.3%, respectively, p > 0.05). No significant difference in the serum ADMA concentration between the NAFLD and control groups was observed (mean: 0.8 ± 0.07 μmol L^-1^ vs. 0.74 ± 0.2 μmol L^-1^, respectively). The CVR profile was significantly higher in the NAFLD group than in the control group (mean: 9% ± 6.9% vs. 4.6% ± 3.8%, P = 0.000). MS associated with NAFLD significantly increased the CVR profile (mean: 11.2% ± 7.4%, P = 0.000). An abnormal serum alanine aminotransferase level (>37 IU L^-1^) and the presence of fibrosis did not increase the CVR profile (p > 0.05).

**Conclusions:**

The risk of cardiovascular events is increased in patients with NAFLD. The association with MS is further increased such risk.

## Background

Non-alcoholic fatty liver disease (i.e. NAFLD) is a common clinical condition of hepatic steatosis in the absence of a history of significant alcohol use or other known liver disease [1–5]. NAFLD is the most common cause of chronic liver diseases in Western countries and also in Turkey [2, 3]. NAFLD encompasses a histological spectrum, ranging from simple steatosis to steatohepatitis, fibrosis, cirrhosis and hepatocellular carcinoma [3]. NAFLD is frequently associated with obesity, type 2 diabetes mellitus (i.e. DM), dyslipidemia, hypertension, metabolic syndrome (i.e. MS) and cardiovascular disease (i.e. CVD) [6, 7]. NAFLD is the hepatic expression of MS [2–5].

Asymmetric dimethylarginine (i.e. ADMA) is an endogenous analogue of L-arginine that may interfere with nitric oxide (i.e. NO) metabolism [5]. ADMA competitively inhibits the activity of NO synthase [7]. Vascular dilatation response is primarily mediated by NO released from arterial endothelial epithelium. The degree of the response correlates with coronary endothelial function. The elevated plasma ADMA concentration was associated with decreased brachial flow-mediated vasodilatation (i.e. FMV) response in healthy adults. Several factors including advanced age, smoking, hyperlipidemia, hypertension, IR, are associated with endothelial dysfunction and CVD [8]. Data regarding the relationship of serum ADMA level, endothelial dysfunction and the potential cardiovascular risk (i.e. CVR) in patients with NAFLD are limited. The aim of the present prospective study were to evaluate the association of the serum ADMA level, endothelial function and the CVR profile in individuals with NAFLD, and to determine whether or not an association with MS affects these parameters.

## Methods

### Patients

This was a prospective longitudinal cohort study. A total of 100 consecutive patients (54 male and 46 female; mean age: 47.8 ± 8.7 years) diagnosed with NAFLD, who were seen in the Liver Diseases Outpatient Clinic were included into the study. Data were obtained from patient visit charts. Criteria for inclusion were: 1) age > 16 years; 2) convincing evidence of absent or minimal alcohol consumption: <20 g alcohol/day for women and <30 g alcohol/day for men; 3) absence of confounding disease including acute and/or chronic viral hepatitis (hepatitis A, B or C); and 4) exclusion of other forms of liver disease including autoimmune, drug-induced and metabolic liver diseases. Diagnosis of NAFLD was based on biochemical, radiological and histological criteria, when available and on exclusion of other forms of acute and chronic liver diseases. None of the NAFLD patients had evidence of cirrhosis. Forty-five healthy controls (23 male and 22 female; mean age: 45.2 ± 7.4 years) were included into study. Hepatitis were excluded by examining all necessary indicators such as serum serum HBsAg, HCV and Anti Hbs levels and also further diagnostic tests if needed. The controls were selected from among individuals with normal clinical included body mass index (i.e. BMI), waist circumference (i.e. WC), and blood pressure, biochemical tests included fasting glucose, lipid profile, aminotransferases levels, and radiological parameters included sonographic examination. Smoking, current pharmacological treatment for comorbid disorders and family history of CVD and DM were evaluated.

Liver biopsy specimens were evaluated by a pathologist that was blinded to the patients’ characteristics. Histological features of the specimens were evaluated according to Brunt et al. [9]. The NAFLD activity score (i.e. NAS) was calculated based on the criteria of Kleiner et al. [6].

MS was diagnosed based on the presence of ≥3 of the following Third Adult Treatment Panel of the National Cholesterol Education Program (i.e. NCEP-ATP III) criteria [10]. (1) fasting glucose ≥100 mg dL^-1^ or history of treatment for diabetes; (2) central obesity (WC >102 cm (males) and >88 cm (females); (3) arterial pressure ≥130/85 mmHg or history of pharmacologically treated hypertension; (4) triglyceride level ≥150 mg dL^-1^ or current use of fibrates; (5) high-density lipoprotein (i.e. HDL) cholesterol ≤40 mg dL^-1^ (males) and ≤50 mg dL^-1^ (females). This study was approved by local ethical committee of Ankara University School of Medicine.

### Methods

Blood pressure measurements were obtained by trained medical assistants, according to the guidelines of the International Society of Hypertension [11]. In all, 3 blood pressure readings were obtained at 1-min intervals; the 2nd and 3rd systolic and diastolic pressure readings were averaged. Body weight was measured to the nearest 0.5 kg with the participants wearing light clothing. Height was measured to the nearest 0.5 cm. The BMI was calculated as weight in kilograms divided by height in meters squared. WC was measured to the nearest 0.5 cm at the shortest point between the lower rib margin and the iliac crest.

Fasting glucose, cholesterol, triglycerides, serum alanine aminotransferase (i.e. ALT), aspartate aminotransferase (i.e. AST), gamma glutamyl transpeptidase (i.e. GGT), alkaline phosphatase (i.e. ALP), bilirubin, and complete blood cell count were measured in our central laboratory using a chemical analyzer and standard reagents. Insulin was measured via radioimmunoassay (i.e. RIA). Insulin resistance (i.e. IR) was calculated on the basis of fasting plasma glucose and insulin values using the homeostasis model assessment-insulin resistance method (HOMA-IR: plasma glucose (mg/dl) × insulin (μu/ml)/405) [12]. For the exclusion of other forms of liver disease serum iron, ferritin, copper, and ceruloplasmin levels were measured, and serological studies for anti-nuclear antibody, anti-smooth muscle antibody, and anti-mitochondrial antibodies were performed. Blood samples for ADMA measurement were centrifuged and the plasma was stored at -80°C until analysis. ADMA was determined using a validated ELISA kit (DLD Diagnostika GMBH, Hamburg, Germany).

### Brachial flow mediated vasodilatation

Brachial FMV was measured by an experienced cardiologist using a high-resolution external ultrasound machine (Vivid 7, Dimension GE, Healthcare, GE Hortan, Norvay) in a quiet room following an overnight fast, according to Celermajer et al. [13]. After identification of the brachial artery, its diameter was measured under 3 conditions: at baseline, in response to shear stress (to induce endothelium-dependent flow mediated dilatation) during hyperemia, and in response to a 400-μg sublingual dose of nitroglycerin. To obtain a high quality baseline measurement ≥10 min of rest in the supine position was needed. Reactive hyperemia was induced via inflation of a sphygmomanometer cuff to 250 mmHg, followed by deflation. After 10–15 min of rest, nitroglycerin was given and 3.5-4 min later a final scan of the atrium was obtained.

The time required to scan is 15 s before and 60 s after cuff deflation. Physiologically, increased blood flow stimulates the release of vasodilators such as NO from the endothelium, which in turn causes arterial dilatation (i.e. FMV); impaired FMV is observed in the presence of endothelial dysfunction. In contrast, nitroglycerin acts directly on arterial smooth muscle and induces endothelium-independent dilatation (i.e. EID). Data were recorded as absolute diameter (mm) and as the percentage of change in diameter after hyperemia and nitrate, respectively.

### Statistical analysis

Data were analyzed using SPSS v.15.0 for Windows. Patients were grouped according to the presence of MS. Values are expressed as mean **±** SD for continuous variables and median (range) for non-normal values. For parametrically distributed data comparisons between the groups were made using the *t*-test and ANOVA. For non-parametrically distributed data the Kruskal Wallis test or Mann–Whitney *U* test was used. The level of statistical significance was set at P < 0.05. Pearson correlation analysis was done to evaluate the relationship between variables.

## Results

The characteristics of the NAFLD patients and controls are shown in Table 1. Two groups were matched for age, gender and cigarette smoking (p > 0.05). Family history of DM and CVD were more prevalent in patients with NAFLD. At the time of the baseline characteristics, the median age of the NAFLD patients was 46.9 years (range 25-63years), BMI was 28.6 (range 19.6-39.7 kg/m^2^); 28% (n = 28) of the patients were obese, 67% (n = 67) of the patients had dyslipidemia, 11% (n = 11) hypertension, 7% (n = 7) DM and 55% (n = 55) IR (HOMA score ≥2.7). Median serum AST, ALT and GGT levels were 38.7 U/L (range: 15–187 U/L), 57.5 U/L (range: 12–248 U/L), and 56.8 U/L (range: 14–312 U/L), respectively. Median initial HOMA score was 3.6 (range: 0.7-24.8), and 50% (n = 50) of the NAFLD patients had MS.

**Table 1:** **Clinical, anthropometric, and biochemical parameters in the NAFLD and control groups (mean ± SD)**

	NAFLD (n = 100)	Control (n = 45)	P
**Age (years)**	47.8 ± 8.7	45.2 ± 7.4	>0.05
**Female/Male**	46/54	22/23	>0.05
**Family history of cardiovascular disease (%)**	17.2 (n = 17)	11.1 (n = 5)	>0.05
**Family history of diabetes (%)**	26.8 (n = 26)	13.3 (n = 6)	>0.05
**Cigarette smoking (%)**	29 (n = 29)	44.4 (n = 20)	>0.05
**BMI (kg m** ^**-2**^ **)**	30 ± 3.9	25.5 ± 2.5	0.000
**Waist circumference (cm)**	95.9 ± 9.2	82.6 ± 5.9	0.000
**Systolic pressure (mmHg)**	117.1 ± 15.0	110.3 ± 9.5	0.001
**Diastolic pressure (mmHg)**	73.9 ± 9.5	69.4 ± 8.3	0.006
**Fasting glucose (74–106 mg dL** ^**-1**^ **)**	90.9 ± 13.6	84.7 ± 8.1	0.006
**Fasting insulin level (4–16 mIU /mL)**	15 ± 10.3	4.2 ± 2	0.002
**HOMA-IR**	3.6 ± 3.1	0.95 ± 0.36	0.000
**Total cholesterol (<200 mg dL** ^**-1**^ **)**	200.8 ± 46.6	171.8 ± 28.1	0.000
**HDL cholesterol (40–60 mg dL** ^**-1**^ **)**	45.4 ± 10.8	51.9 ± 11.3	0.001
**Triglycerides (<150 mg dL** ^**-1**^ **)**	167.6 ± 103.7	95.3 ± 34.2	0.000
**Aspartate aminotransferase (<31 IU mL** ^**-1**^ **)**	38.7 ± 25.2	19.3 ± 4.8	0.000
**Alanine aminotransferase (<31 IU mL** ^**-1**^ **)**	57.5 ± 42.6	18 ± 7.8	0.000
**ADMA (** **μmol L** ^**-1**^ **)**	0.8 ± 0.07	0.74 ± 0.2	>0.05

BMI; body mass index, HOMA-IR: Homeostasis model assessment-insulin resistance, HDL; high density lipoprotein, ADMA; Asymmetric dimethylarginine.

### Endothelial function

**Figure 1:**
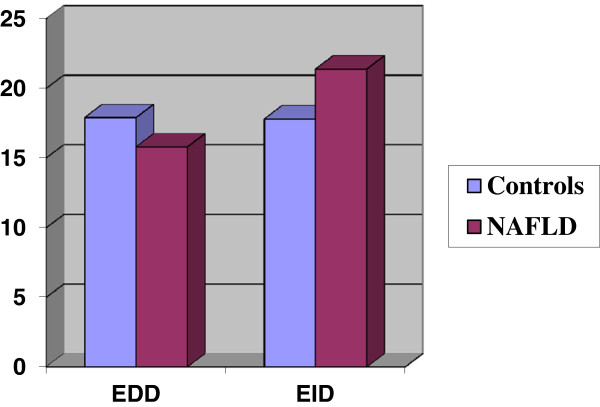
**EDD and EID percentages in the NAFLD and control groups.**

At baseline, no significant difference in term of the diameter of the brachial artery between NAFLD patients and controls was observed (3.7 ± 0.6 mm vs. 3.6 ± 0.6 mm, respectively). There was no significant difference in the serum ADMA concentration between two groups observed (mean: 0.8 ± 0.07 μmol L^-1^ vs. 0.74 ± 0.2 μmol L^-1^, respectively). FMV and flow-independent vasodilatation in response to sublingual nitroglycerin did not differ between the NAFLD patients and controls (mean: 16 ± 9.4% vs. 17.9 ± 12.4%, and 21.4 ± 14% vs. 17.8 ± 11.3%, p > 0.05, respectively) (Figure 1).

In patients with NAFLD, 62% of the patients had an abnormal serum ALT level (mean: 76 ± 43 U/L, <31 IU mL^-1^). There was no significant association between elevated serum ALT levels, and FMV or flow-independent vasodilatation (mean: 16.2% ± 10.6%, 15% ± 6.9%, and 22.2% ±16.3%, 20.1% ± 9.8%, respectively). Additionally, there wasn’t a significant difference detected with MS or fibrosis or both of them.

### CVR profile

The 10-year probability of cardiovascular events was evaluated according to the Framingham equation and the CVR scores were categorized as low (<10%), intermediate (10%-20%), and high (>20%) [14] (Table 2). CVR was significantly increased in patients with NAFLD as compared to controls (mean: 9.0% ± 6.9% vs. 4.6 ± 3.8%, respectively, p < 0.001). In patients with NAFLD, the association with MS was significantly higher the CVR (mean: 11.2% ± 7.4%, p < 0.001). However, abnormal serum ALT level and the presence of hepatic fibrosis were not associated with an elevated CVR profile (p > 0.05).

**Table 2:** **Cardiovascular risk assessment in the NAFLD and control groups**

	<10% (low)	10%-20% (intermediate)	>20% (high)
**Controls (%) (n = 45)**	88.6	11.4	0
**NAFLD (%) (n = 50)**	80	16	4
**NAFLD and MS (%) (n = 50)**	48	40	12

NAFLD; Non-alcoholic fatty liver disease, MS; metabolic syndrome.

EDD: Endothelium-dependent dilatation.

EID: Endothelium-independent dilatation.

We analysed correlation between variables and only found significant positive correlation only between fibrosis and fasting insulin, HOMA score and waist circumference (p = 0.001, r = 0.63, p = 0.001, r = 0.64, and p = 0.04, r = 0.42, respectively).

## Discussion

In the present study, we investigated that the association of the serum ADMA level, endothelial function and the CVR profile in NAFLD patients, and compared those of age- and sex-matched healthy controls. Endothelial dysfunction is associated with coronary atherosclerosis in the very early stages of disease and is indicative of disease grade [15]. A common approach for assessing endothelial function is brachial artery Doppler ultrasonography. In addition to this non-invasive technique, measurement of plasma levels of markers of endothelial reactivation, such as ADMA can be used. Villanova et al. investigated endothelial function in 52 NAFLD cases and 28 controls, and reported that there was endothelial dysfunction in the NAFLD patients, particularly in patients with non-alcoholic steatohepatitis (NASH), as compared to those of patients with simple steatosis [16]. Senturk et al. confirmed these findings [17]. Kasumov et al. reported that the ADMA level was significantly higher in patients with NAFLD [18]. In the present study, in contrast to previous studies, no significant difference between NAFLD patients and control groups in terms of the diameter of the brachial artery and the serum ADMA concentration was observed (p > 0.05). This difference may be explained that NAFLD patients in the present study had lower serum triglyceride and fasting plasma glucose levels, and a lower HOMA-IR score, and had small number hypertensive and diabetic patients compared to those of previous studies [16, 18].

Colak et al. found higher CIMT levels, and lower FMD measurements in NAFLD group than in the controls. The results were found independent from MS and it was also more evident in patients with simple steatosis and NASH compared to control group [19]. Kim et al. investigated the association between NAFLD and CIMT, according to the presence of MS, and observed a significant difference in CIMT measurements in patients with MS [20].

The Framingham score is extensively used to calculate CVR and was previously shown to correlate with biochemical markers of endothelial function [21]. An increased CVR are associated with advanced age, male gender, high blood pressure, cigarette smoking, dyslipidemia, diabetes, and a family history of CVD [22, 23]. Several studies have suggested that NAFLD is the hepatic manifestation of MS, a condition strictly associated with atherosclerosis [2, 3, 5]. Schwimmer et al. reported that based on clinical observation, patients with NAFLD, who diagnosed in childhood had early-onset atherosclerosis [24]. Moreover, Villanova et al. reported an increased the 10-year probability of cardiovascular events in patients with NAFLD [16]. This finding is consistent with the present study demonstrating CVR was significantly higher in the NAFLD patients than controls, especially among the patients with MS.

The present study has limitations. Firstly, the number of controls are limited. Secondly, it will be better to study other methylated arginines including monomethyl-arginine, (i.e.MMA), and symmetric dimethylarginine (i.e. SDMA) in addition to the ADMA.

In conclusion, based on the results of this study, cardiovascular risk is increased in patients with NAFLD. Association with MS seems to further increase such risk in patients with NAFLD. However, endothelial dysfunction is not found in patients with NAFLD according to the FMD measurements and ADMA levels. Further studies with larger number of patients including determination of other circulating molecules related to the endothelial function is needed to clarify whether endothelial dysfunction contributes to the increased cardiovascular risk or not.

### Consent

Written informed consent was obtained from the patients for the publication of this report.

## Abbreviations

NAFLD: Non-alcoholic fatty liver disease
NASH: Non-alcoholic steatohepatitis
IR: Insulin resistance
MS: Metabolic syndrome
NAS: NAFLD activity score
CIMT: Carotid intima media thickness
HOMA-IR: Homeostasis model assessment-insulin resistance
BMI: Body mass index
WC: Waist circumference
hs-CRP: High-sensitivity C-reactive protein.
